# RhoJ facilitates angiogenesis in glioblastoma via JNK/VEGFR2 mediated activation of PAK and ERK signaling pathways

**DOI:** 10.7150/ijbs.65653

**Published:** 2022-01-01

**Authors:** Mei Wang, Chengfei Zhang, Qian Zheng, Zhijie Ma, Min Qi, Guangfu Di, Shizhang Ling, Haojun Xu, Bin Qi, Chengyun Yao, Hongping Xia, Xiaochun Jiang

**Affiliations:** 1 The Translational Research Institute for Neurological Disorders & Interdisciplinary Research Center of Neuromedicine and Chemical Biology of Wannan Medical College and Anhui Normal University, Department of Neurosurgery, the First Affiliated Hospital of Wannan Medical College (Yijishan Hospital of Wannan Medical College), Wuhu 241001, PR China.; 2 School of Basic Medical Sciences & State Key Laboratory of Reproductive Medicine & Key Laboratory of Antibody Technique of National Health Commission & Jiangsu Antibody Drug Engineering Research Center, Nanjing Medical University, Nanjing 211166, PR China.; 3 Center of Clinical Research, The Affiliated Wuxi People's Hospital of Nanjing Medical University, Wuxi 214023, PR China.; 4 Department of Neurosurgery, The First Hospital of Jilin University, Changchun130021, PR China.; 5 Jiangsu Cancer Hospital & The Affiliated Cancer Hospital of Nanjing Medical University & Jiangsu Institute of Cancer Research, Nanjing 2100092, PR China.; 6 Department of Microbiology and Immunology, Medical School of Southeast University, Nanjing 210009, PR China.

**Keywords:** RhoJ, Angiogenesis, GBM, VEGFR2, JNK

## Abstract

Glioblastoma (GBM) is a highly vascularized malignant tumor that depends on new blood vessel formation. Small molecules targeting the angiogenic process may be an effective anti-GBM therapeutic strategy. We previously demonstrated that RhoJ promoted the progression and invasion of GBM. RhoJ has also been shown to be expressed in endothelial cells and plays an important role in regulating endothelial cell migration and tumor angiogenesis. Therefore, we aimed to evaluate the role and mechanism of actions of RhoJ in GBM angiogenesis. We analyzed the expression of RhoJ in different grade gliomas and investigated its role in GBM angiogenesis* in vivo* and *in vitro*. Furtherly, RNA sequencing (RNA-seq), Western blotting and immunofluorescence were performed to identify the molecular mechanism of RhoJ in regulating endothelial cell behavior and GBM angiogenesis. Here, we found that silencing RhoJ resulted in inhibition of HUVEC cell migration and blood vessel formation. Overexpression of RhoJ promoted the expression of CD31, EpCAM and moesin, suggesting RhoJ facilitated angiogenesis and the malignant progression of GBM. RNA-seq data showed that VEGF/TNF signaling pathway positively regulated RhoJ. The expression levels of RhoJ was upregulated with the stimulation of VEGF, and reduced by the treatment of JNK inhibitor SP600125. It was also found that the activity of PAK-BRAF-ERK was down-regulated upon RhoJ and JNK knockdown. In conclusion, these results suggested that RhoJ plays an essential role in regulating GBM angiogenesis through the JNK/VEGFR2-PAK-ERK signaling pathway and there might exist a VEGF-JNK/ERK-VEGF circuitry. Thus, RhoJ may be a candidate therapeutic target for anti-angiogenesis treatment in GBM.

## Introduction

Glioblastoma (GBM) is the most malignant and fatal primary brain tumor, characterized by rapid proliferation, diffuse infiltration and high vascularization. The progression of GBM depends on angiogenesis, the process of new blood vessel formation from the preexisting ones 1. GBM angiogenesis is mediated by many angiogenic factors and genes. Vascular endothelial growth factor (VEGF) as the best-known mediator contributes to sprout formation, neovascularization and tumorigenesis via stimulation of VEGF receptor 2 (VEGFR2) 2, 3, the main VEGFR (VEGFR1-R3) in blood vascular endothelial cells. Activated VEGFR2 can induce various intracellular signaling molecules, including RAF-MEK-ERK1/2 signaling 4, stress kinase p38 MAPK 5, TSAd-SRC 6 and PI3K-AKT pathways 7, 8. PI3K kinase-dead suppresses endothelial migration, sprouting and vascular remodeling by reducing small GTPase activity 9.

As a part of the small G protein family, the small GTPases of the Rho family regulate actin structure and participate in endothelial cells angiogenic processes, including proliferation, migration, adhesion and survival 10. Phosphorylated VEGFR2 allows activation of Rho family Cdc42 and PAKs (p21-activated kinases, PAKs), the main effector of the Rho GTPases family 11, 12. Yet, the mechanism of VEGFR2 signaling in regulating Rho GTPases during angiogenesis remains to be determined. VEGF-induced endothelial cell migration and tube formation require RhoQ and RhoJ 13, 14. RhoQ/TC10 was proved to mediate VEGFR2-induced GLUT-1 trafficking in mouse embryonic stem cells 15. Esther Bridges et al. demonstrated that RhoQ/TC10 acts as a downstream target of Notch/DLL4 signaling in endothelial cells during angiogenesis by using gene knockout and overexpression studies in different angiogenesis models *in vitro* and* in vivo* 16.

RhoJ (TC10-Like, TCL) belongs to the Rho GTPase Cdc42 subfamily and is highly expressed in endothelial cells, which plays an important role in regulating angiogenesis via different pathways 17. It was reported that the endothelial-specific transcription factor, ERG, regulates the expression of RhoJ mediating vascular morphogenesis 18. Kim et al. used a mouse model to show that blocking RhoJ inhibits tumor angiogenesis by activating the RhoA-ROCK signaling pathway in tumor endothelial cells and destroying the pre-formed tumor blood vessels, ultimately leading to the failure of the tumor vascular system 19.

However, the role and mechanism of actions of RhoJ in endothelial cells during malignant GBM angiogenesis are currently unknown. Here, we explored the effect of RhoJ by gene knockdown or overexpression approaches *in vitro* and *in vivo* and demonstrated a therapeutic potentiality of deficit RhoJ in anti-GBM angiogenesis.

## Materials and methods

### Cell lines, cell culture and treatment

Human umbilical vein endothelial cells (HUVECs) were purchased from Gibco (A1460901) and cultured with medium 200 supplemented with 1× large vessel endothelial supplement (LVES). 293T cells, human GBM cells, U251 and U87, were cultured in a complete DMEM medium and incubated in 37 °C incubators with 5% CO_2_. HUVECs were treated with VEGF165 (PF0602, Cellregen, Beijing, China) or JNK inhibitor SP600125 (HY-1204, MedChemExpress, USA) at indicated concentrations for the indicated time in subsequent experiments. Bevacizumab (HY-P9906) was purchased from MedChemExpress, USA.

### Generation of RhoJ stable knockdown or overexpressing cell lines

RhoJ knockdown or overexpressing lentivirus were obtained from 293T cells transduced with RhoJ targeting shRNA or overexpression plasmids or control vector by lentiviral transduction protocols as previously described 20. HUVECs and GBM cells were infected by the lentivirus as mentioned above and selected with 2 μg/mL puromycin (A610593, Sangon Biotech, Shanghai, China) for about two weeks to obtain stably transfected cell lines.

### Animal experiment

Stable expressing plenti-con or plenti-RhoJ U87 cells (5×10^6^) were subcutaneously injected into the flank of male NOD-SCID mice (6-8 weeks old, n=4). Tumor size was measured every four days and tumor volume was calculated by the formula: (length × width^2^)/2. About one month later, mice were euthanized and xenografted tumor tissues were collected for weighing and subsequent immunohistochemical staining analysis.

### Immunohistochemical (IHC) staining

The normal brain tissue paraffin sections were obtained from the Department of Forensic Medicine, Nanjing Medical University and the tissue sections of Grade II to Grade IV glioma were gifts from the Department of Neurosurgery, Yijishan Hospital of Wannan Medical College. The samples, as mentioned earlier, were stained with primary antibody against RhoJ (H00057381-M01, Abnova, Taiwan, China) at 1:100 dilution. The collected xenograft tumor tissues were embedded and sectioned (5 µm), then stained with anti-CD31 antibody (A18643, Abclonal, Wuhan, China) at 1:50 dilution, anti-RhoJ antibody (H00057381-M01, Abnova, Taiwan, China) at 1:100 dilution, anti-moesin antibody (M7060, Sigma, USA) at 1:100 dilution, or anti-EpCAM antibody (ab187372, Abcam, United Kingdom) at 1:100 dilution.

### Immunofluorescent staining (IF)

The collected xenograft tumor tissues were fixed with 4% paraformaldehyde (PFA), gradient dehydration in a series of increasing concentrations of sucrose, then embedded in optimal cutting temperature (OCT) compound (4583, Sakura, Japan) and sectioned at 20 µm thickness for IF staining. The sections were rinsed with 0.3% Triton X-100 in PBS (PBST) for 5 min thrice, blocked with 10% fetal bovine serum (FBS, Gibco, USA) in 0.3% PBST at room temperature (RT) for 1.5 h, then incubated with EpCAM (ab187372, Abcam, United Kingdom) at 1:100 dilution and anti-CD31 (A18643, Abclonal, Wuhan, China) at 1:50 dilution at 4 °C overnight. After washing for 3 times, the slides were incubated with a corresponding secondary antibody at RT for 2 h. After washing for 3 times, the samples were incubated with 4′,6-diamidino-2-phenylindole (DAPI, Sigma, USA) at RT for 30 min. The cell immunofluorescent staining steps were similar to the IF of tissue sections above. The rhodamine-conjugated phalloidin (40734ES75, Yeasen, China) was stained for 30 min before DAPI.

### Transwell migration assay and tube formation assay

The cellular migration ability was measured by a transwell assay using chambers with transwell inserts of 8 µm in pore size in a 24-well plate. 5×10^4^ cells suspended in 200 µL normal culture medium were seeded in the upper chamber for HUVECs with shNC (negative control shRNA), shRhoJ (RhoJ targeting shRNA), shRhoJ+con (shRhoJ combined with plenti-control), or shRhoJ+oe (shRhoJ combined with plenti-RhoJ), medium 200 supplemented with 20 ng/ml VEGF was placed in the lower chamber. For the U87 and U251 transwell assay, the conditioned medium (CM) of HUVECs-shNC or HUVECs-shRhoJ was placed in the lower chamber. 24 h after seeding, cells were fixed with 4% PFA and stained with 1% crystal violet staining solution.

The angiogenesis ability of HUVECs was conducted by* in vitro* tube formation assay. LDEV-Free Reduced Growth Factor Basement Membrane Matrix (A14132-02, Gibco, USA) was thawed at 4 ℃ overnight. The next day, Matrix was placed on a pre-chilled 24-well plate and then coagulated at 37 ℃ for 30 min. 1×10^5^ cells/well were seeded in the Matrix coated plate and cultured for 16-18 h, then the cells were fixed and photographed under a light microscope.

### Spheroid sprouting assay

HUVEC spheroids (~800 cells/drop) were generated by the previously described hanging drop method 21. Briefly, HUVECs were cultured in hanging drops in medium 200 overnight. Then the spheroids were collected and embedded in Growth Factor Reduced (GFR) Matrigel (354230, Corning, USA). 50 ng/mL VEGF was added to induce the sprouting of HUVECs. After incubation for 48 h, spheroids were imaged under a light microscope.

### RNA isolation and sequencing

Total RNA from U251-shNC and U251-shRhoJ was isolated by Trizol (15596026, Invitrogen, USA), the quality and quantity of RNA samples were measured using a NanoDrop 2000 spectrophotometer (ThermoFisher Scientific, USA), then sent to Novogene Company (Beijing, China) for RNA-seq. 5 μg of total RNA (RNA integrity number ≥7) per sample was prepared for the generation of sequencing libraries. Oligo (dT) magnetic beads were used to bind poly(A) tail structures in mature mRNAs in eukaryotes to remove rRNAs and other RNAs. Then fragmentation buffer was added to segment the purified mRNA into small fragments. Double-stranded cDNA (dscDNA) were synthesized using the mRNA fragments as templates. First-strand cDNA was synthesized by reverse transcriptase using random primers. To form a double-stranded structure, the second strand cDNAs were synthesized and purified. Then the End Repair Mix was used to patch a cohesive end into a blunt end. DNA beads were used in the purified dscDNA to screen 200-300 bp bands. Sequencing libraries were prepared using NEBNext Ultra II DNA Library Prep Kit for NGS performed on HiSeq-PE150 (Illumina, USA) platform, with each sample typically sequenced to 10-30 million read lengths.

After obtaining the raw data from RNA-Seq, firstly, we need to evaluate the quality of raw data, clean reads filtering and remove untrusted data from raw data to obtain high-quality clean data. Then, the differently expressed genes (DEGs) analysis was performed using the 'DEseq2' R package (|log 2 FC|>0.5 and FDR < 0.05). Heatmaps were centered and scaled in the row direction. GO (Gene Ontology, http://www.geneontology.org/) was also performed.

### Quantitative RT-PCR

The total RNA was reverse-transcribed using the 5× All-In-One RT MasterMix (Applied Biological Materials Inc. ABMgood, Zhenjiang, China). cDNA amplification was performed using qPCR SYBR® Green Master Mix (Yeasen, Shanghai, China). Each assay was implemented in triplicate. The primer sequences are listed in Supplementary Table 1.

### Immunoblotting assay

Cells were lysed in RIPA buffer (Beyotime Biotechnology, China) containing protease and phosphatase inhibitors, and the total protein was quantified by BCA assay (Beyotime Biotechnology, China). The subsequent Western blotting analysis was carried out according to the usual procedure 22. The primary antibodies used are listed as follows: p-VEGFR2-Y1175 (AP0382, Abclonal, China); Tubulin (AC008, Abclonal, China); p-JNK1/2/3 (AP0631, Abclonal, China); JNK1/2/3 (A11119, Abclonal, China); p-PAK2(Ser20) (AP0803, Abclonal, China); p-PAK4(Ser474) (3241S, Cell Signaling Technology, USA); RhoJ (H00057381-M01, Abnova, Taiwan, China); moesin (M7060, Sigma, USA); GAPDH (A19056, Abclonal, China); β-actin (AC026, Abclonal, China); Braf (14814, Cell Signaling Technology, USA); p-Braf (Ser445) (2696, Cell Signaling Technology, USA); ERK1/2 (9102, Cell Signaling Technology, USA); p-ERK1/2 (4370, Cell Signaling Technology, USA).

### CCK8 assay

3000 HUVECs per well were seeded in a 96-well plate and treated with VEGF (100 ng/mL) or SP600125 (5 µM) or the combination of these two drugs for 0 h, 12 h, 24 h, or 48 h. The cell viability was evaluated by CCK-8 kit (40203ES60, Yeasen, China), the optical absorbance at 450 nm was measured using the Tecan Spark microplate reader (Switzerland).

### Statistical analysis

Statistical analyses were carried out with GraphPad Prism 5 (GraphPad Software). The two-tailed Student's t-test was used to define statistical significance between groups. All data were presented as mean ± SEM (standard error of mean). p< 0.05 was considered statistically significant.

## Results

### Overexpression of RhoJ promotes GBM progression and angiogenesis* in vivo*

**Figure 1:**
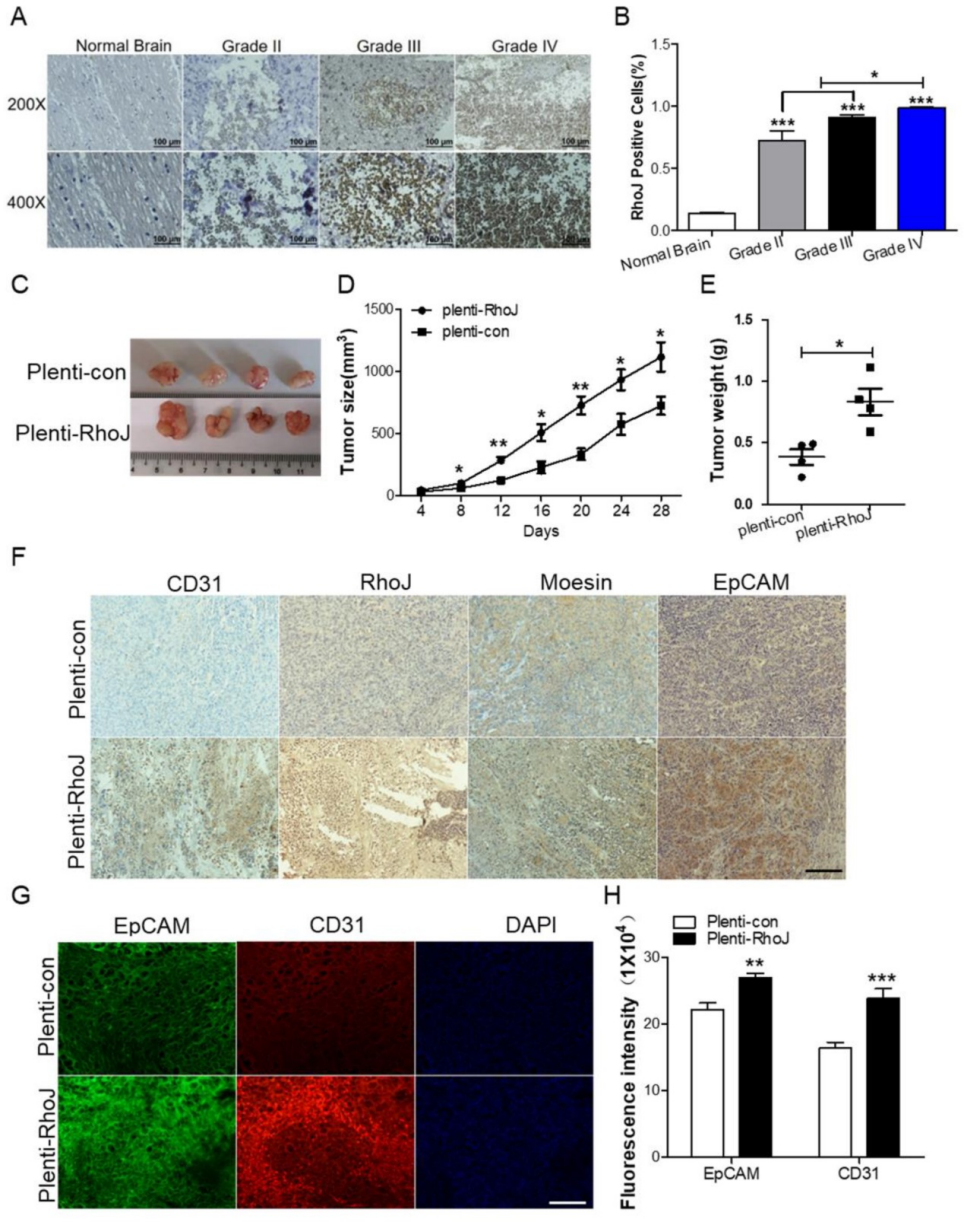
Overexpression of RhoJ promotes GBM progression and angiogenesis in the xenograft mouse model. (A) Representative images of RhoJ expression in the normal brain and grade II to grade IV glioma tissues by IHC staining, obtained at 200× magnification (upper) and 400× magnification (lower). All scale bars, 100 μm. (B) Semi-quantitative analysis results of RhoJ positive cells in different grades of glioma (WHO II-IV) and normal brain tissue samples by immunohistochemical (IHC) staining. (C) Representative tumor xenografts from U87 cells expressing plenti-RhoJ or plenti-con. (D) The xenograft tumor size was measured every 4 days before they were sacrificed. (E) The xenograft tumor weight was measured after sacrifice (mean ± SEM, n = 4). (F) IHC analyses of CD31, RhoJ, meosin and EpCAM in the xenograft tumor. scale bar, 100 μm. (G) Representative images of tumor xenografts for IF analysis of EpCAM and CD31. Scale bar, 100 μm. (F) Quantitative analysis of fluorescence intensity of EpCAM and CD31 in (G). Scale bar, 100 μm. In all images, data are shown as the mean ± SEM. Every experiment was independently repeated 2-3 times. *p<0.05; **p<0.01; ***p<0.001.

In our previous study, we found that RhoJ was upregulated in GBM tissues and associated with poor patients' survival and upregulated RhoJ promoted the invasion and progression of GBM 20. Considering the importance of angiogenesis in the progression of GBM, we further investigated the role of RhoJ in GBM angiogenesis. In this study, we firstly verified that the expression of RhoJ was positively correlated with the malignancy of glioma. RhoJ level increased with a higher grade of human brain gliomas by IHC analysis **(Fig. 1A, B)**. To study the effect of RhoJ on GBM angiogenesis and growth* in vivo*, we subcutaneously injected U87 cells infected by plenti-RhoJ or plenti-con in NOD-SCID mice and found that the tumor tissues in plenti-RhoJ group grew faster, larger and heavier, compared with those in plenti-con group **(Fig. 1C-E)**. We then collected the xenograft tumor tissues for the paraffin and frozen sectioning, respectively. Both IHC and IF staining results showed that the expression of CD31, RhoJ, EpCAM, and moesin were significantly increased in tumor tissues derived from the RhoJ-overexpressing group** (Fig. 1F, G)**. All these data indicated that RhoJ could promote GBM angiogenesis and tumor growth* in vivo*.

### Silencing RhoJ inhibits endothelial cell migration and tube formation *in vitro*

**Figure 2:**
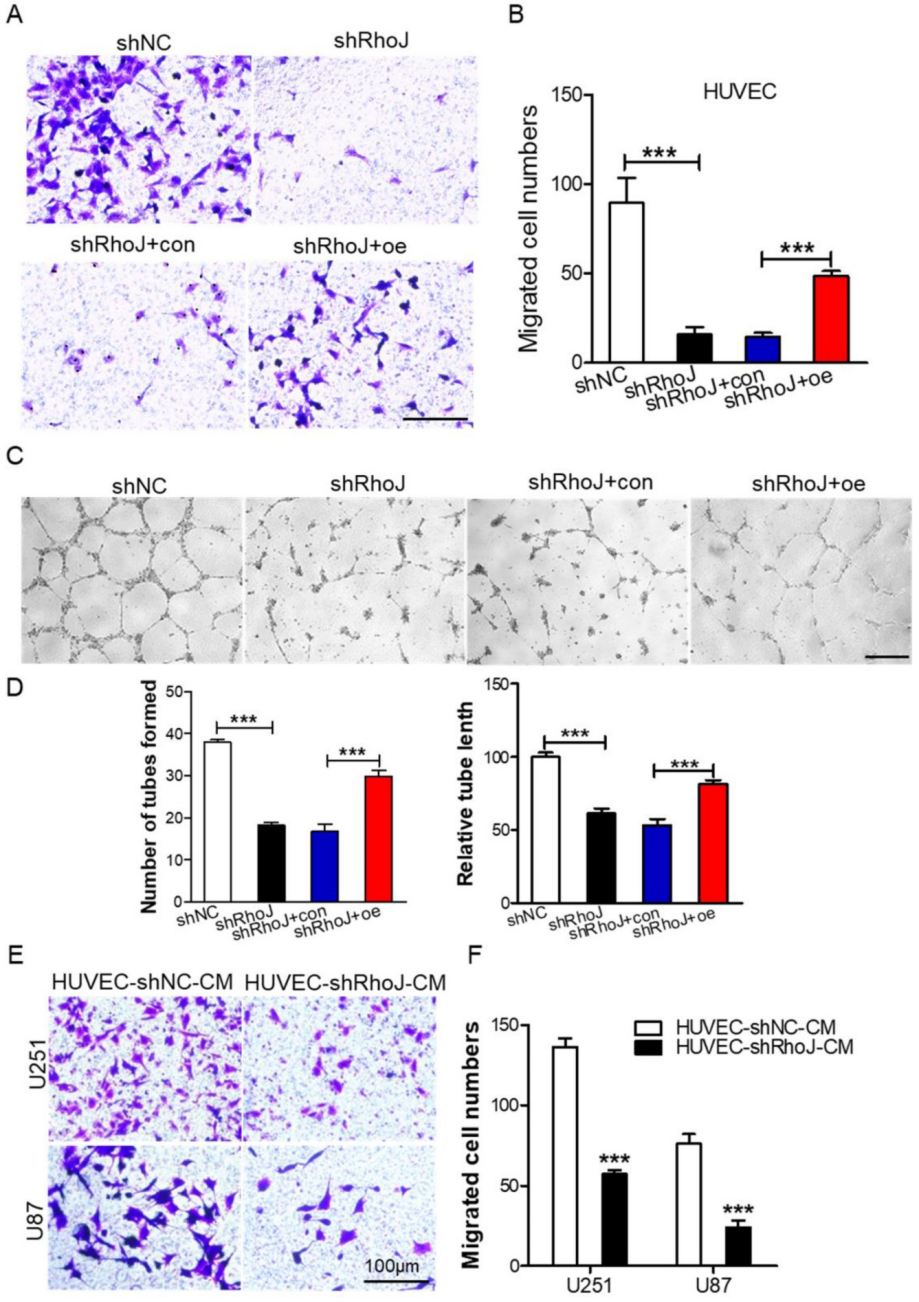
Silencing RhoJ inhibits endothelial cell migration and tube formation *in vitro*. (A) Represented images of migrated HUVECs with shNC, shRhoJ, shRhoJ+con, or shRhoJ+oe by transwell assay. Scale bar, 100 μm. (B) Migrated cells were measured by a transwell assay. (C) Tube formation assay detected the formed tubes of HUVECs with shNC, shRhoJ, shRhoJ+con, or shRhoJ+oe. Scale bar, 100 μm. (D) The number of tubes formed and tube length were calculated to quantify the ability of tube formation (shRhoJ group vs shNC group; shRhoJ+ RhoJ-oe group vs. shRhoJ+con group). (E) Migrated cells of U87 and U251 cells treated with CMs from HUVECs expressing shRhoJ or shNC were measured by transwell assay. Scale bar, 100 μm. (F) Quantitative analysis of the migrated U251 and U87 cells. Data are shown as the mean ± SEM. Every experiment was repeated three times. ***p<0.001.

Endothelial cell migration is a key step in the process of tumor angiogenesis 23. Firstly, after we concentrated the packaged shNC, shRhoJ and RhoJ-oe lentiviruses, we infected HUVECs with these lentiviruses, and then screened stably transfected cells by puromycin. After the stably transfected cells were obtained, then transwell migration assay was performed to measure the role of RhoJ in HUVECs chemotactic motility. We found that RhoJ silencing inhibited HUVECs migration while RhoJ overexpression increased the migration ability **(Fig. 2A, B)**. Furthermore, the tube formation assay was performed and the results showed impaired tubular structures, fewer numbers of the tube formed, and shorter tube length in the shRhoJ group compared to shNC group. This phenotype can be rescued by overexpressing RhoJ **(Fig. 2C-D)**. Transwell assay was used to observe the influence of conditioned medium (CM) from HUVECs on GBM cell migration ability. Data showed that the number of migrated U87 and U251 cells in the supernatant medium in the HUVEC-shRhoJ group was significantly lower than that of the control group, suggesting that RhoJ silencing in HUVEC cells inhibited the migration of GBM cells** (Fig. 2E-F)**. Furthermore, the CM from U87-shRhoJ cells also inhibited the migration and tube formation ability of HUVECs (**Fig. S1 A-D**). We measured the secreted VEGF from U87 and HUVECs by ELISA and found that knockdown of RhoJ significantly decreased the VEGF secretion from U87 and HUVEC cells (**Fig. S1 E-F**). These results showed that RhoJ could encourage HUVECs migration and tube formation and HUVECs mediated the migration of GBM cells.

### Depletion of RhoJ impairs the TNF signaling pathway

**Figure 3:**
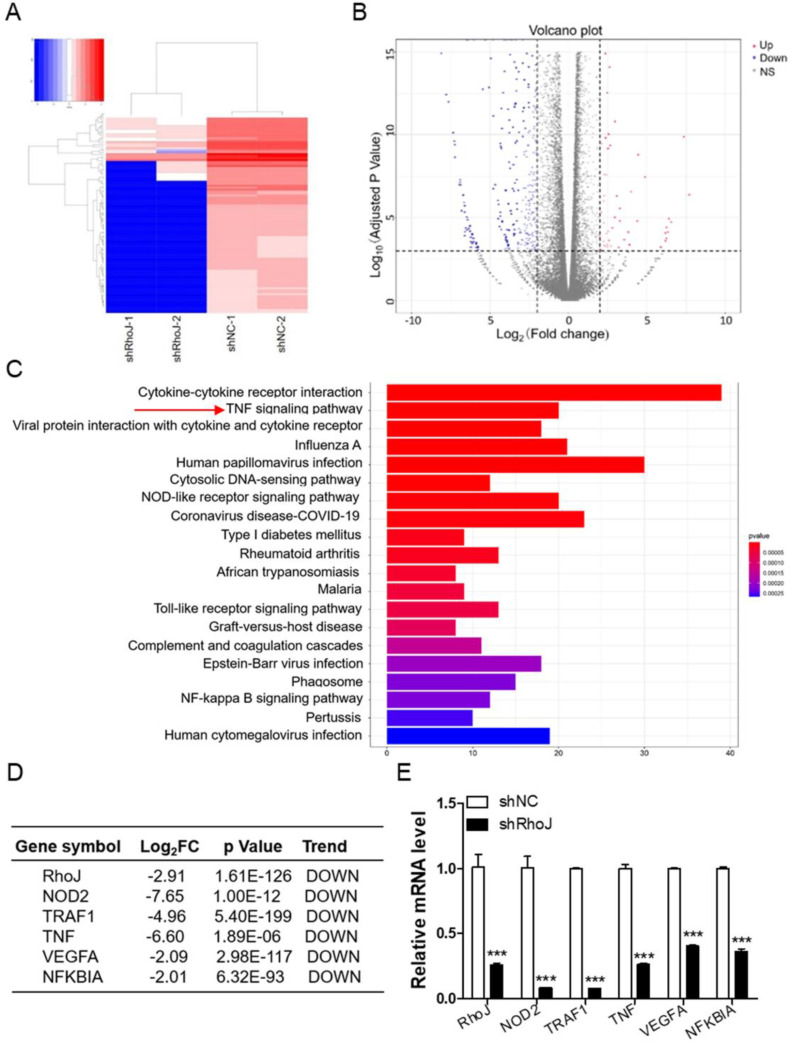
Depletion of RhoJ impairs the TNF signaling pathway. (A) Heatmap of the top 100 most differentially expressed genes (DEGs). The color change from blue to red represents the changing process from down-regulation to up-regulation. (B) A volcano plot of DEGs from RNA sequencing is shown. (C) GO biological pathway analysis of the down-regulated genes—cell signaling pathways. (D) List of the fold change of the selected down-regulated genes RhoJ, VEGFA and TNF-related genes NOD2, TRAF1, TNF and NFKBIA from RNA sequencing data. (E) qPCR validation of the selected genes. Data are shown as the mean ± SEM. Every experiment was repeated three times. ***p<0.001.

To determine the mechanism of RhoJ in GBM angiogenesis, we performed an RNA sequencing using U251 cells to profile differentially expressed genes (DEGs) and further determine the pathways associated with the loss of RhoJ. As shown in the data from hierarchical clustering analysis of the top 100 most DEGs, knockdown of RhoJ mainly decreased the levels of most RNAs **(Fig. 3A)**. And volcano plot represented a total of 802 DEGs identified in the shRhoJ group compared with the control group, including 692 downregulated genes and 110 upregulated genes **(Fig. 3B)**. We further performed GO (Gene Ontology) analysis based on this gene list to disect the pathways involved. We found that the decrease of RhoJ dramatically downregulated the TNF signaling pathway and other inflammation and immune-related pathways **(Fig. 3C)**. Here, we focused on the TNF pathway and listed the related various genes, such as NOD2, TNF, TRAF1, NFKBIA. In addition, VEGFA, which was involved in tumor angiogenesis, was also downregulated **(Fig. 3D)**. We then validated these genes by qPCR and found that the loss of RhoJ significantly decreased the expression levels of these genes in GBM cells **(Fig. 3E)**.

### Blockade of JNK inhibits VEGF-induced HUVECs behavior

**Figure 4:**
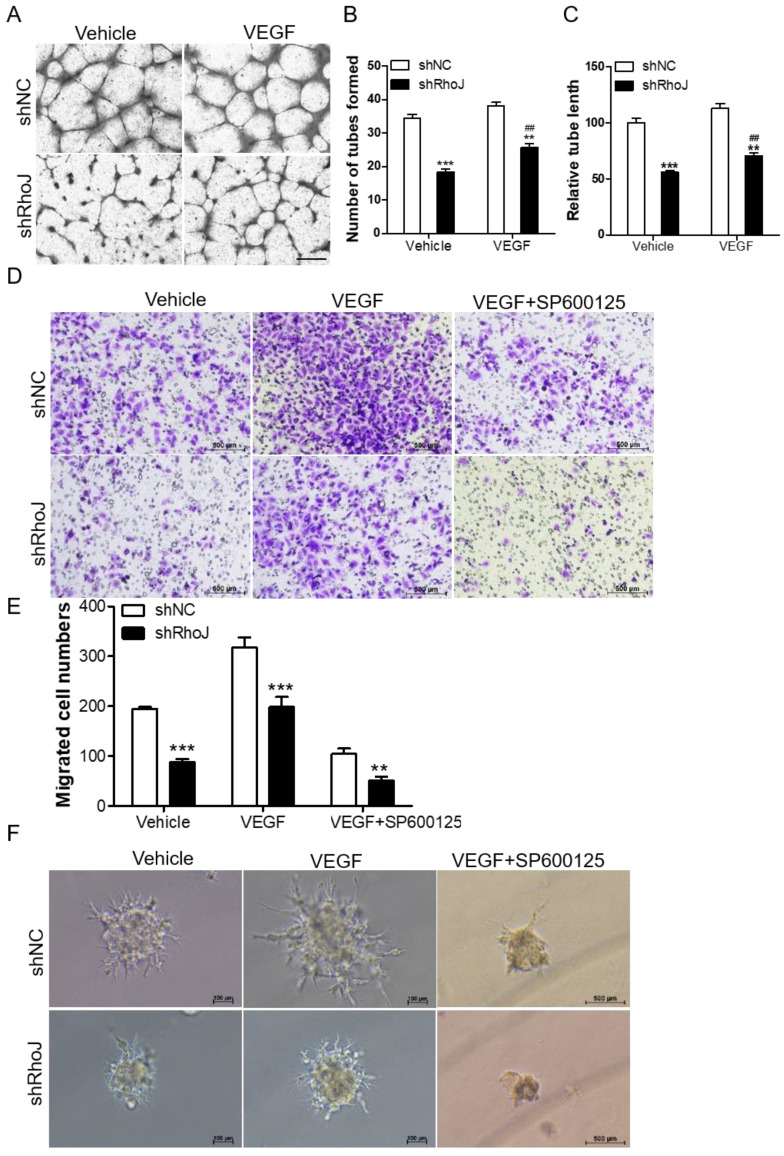
Blockade of JNK inhibits VEGF-induced HUVECs behavior. (A) Tube formation assay detected the formed tubes of HUVECs with shRhoJ or shNC treated by vehicle or VEGF (50 ng/ml). Scale bar, 100 μm. (B) The number of tubes formed and (C) tube length were calculated to quantify the ability of tube formation (*: shRhoJ group vs. shNC group; #: VEGF group vs. vehicle group). (D) Representative images and (E) graphical representation of transwell migration assay of HUVECs expressing shNC or shRhoJ treated by Vehicle, VEGF or JNK inhibitor SP600125. Scale bar, 50 μm. (F) Representative images of phenotypic changes in the hanging drop sprouting assay, HUVECs were expressing shNC or shRhoJ treated by Vehicle, VEGF, or JNK inhibitor SP600125. Scale bars, 50 μm. Data are shown as the mean ± SEM. Every experiment was repeated three times. ^##^ p<0.01; **p<0.01; ***p<0.001.

VEGF is an important factor in promoting angiogenesis. The binding of VEGF to VEGFR activated its downstream pathways to promote proliferation and migration of vascular endothelial cells, which led to the malignant progression of tumors. VEGFR mainly consists of three members, VEGFR1, VEGFR2 and VEGFR3. Among them, VEGFR2 is a key factor in mediating the neovascularization and permeability of VEGF 24. We stimulated HUVECs with RhoJ stable knockdown or control HUVECs with VEGF (50 ng/mL) for 8 h, or treated the HUVECs-con or HUVECs-RhoJ-oe with 5 μg/ml Bevacizumab (BEV, Avastin), a monoclonal antibody against VEGF, then performed a tube formation assay to verify the effect of RhoJ on angiogenesis. On the one hand, loss of RhoJ significantly reduced the number of tubes and the total length of endothelial cell branches compared with the control group, and VEGF stimulation further promoted the formation of endothelial tubes in shRhoJ-cells **(Fig. 4A-C)**. On the other hand, overexpression of RhoJ promoted the migration and tube formation ability of HUVECs, while RhoJ function was inhibited by anti-VEGF antibody BEV (**Fig. S2 A-D**).

TNF signaling triggers the activation of many pathways, including the NFκB, JNK and MAPK pathways 25, 26. Additionally, our previous research reported that RhoJ was directly regulated by c-Jun, the downstream effector of JNK kinase. In this study, we found that VEGF increased the migration ability of RhoJ-knockdown HUVECs, while SP600125, a highly selective inhibitor of JNK, inhibited VEGF-induced migration ability of HUVECs (**Fig. 4D, E**). Consistent with these findings, in the hanging drop sprouting assay, disruption of RhoJ significantly reduced the number and the length of sprouts of HUVECs spheroids, and VEGF-induced hyper-sprouting phenotype was inhibited by SP600125 treatment (**Fig. 4F**).

### JNK/JNK/VEGFR2/RhoJ mediates the activation of PAK and ERK pathways

**Figure 5:**
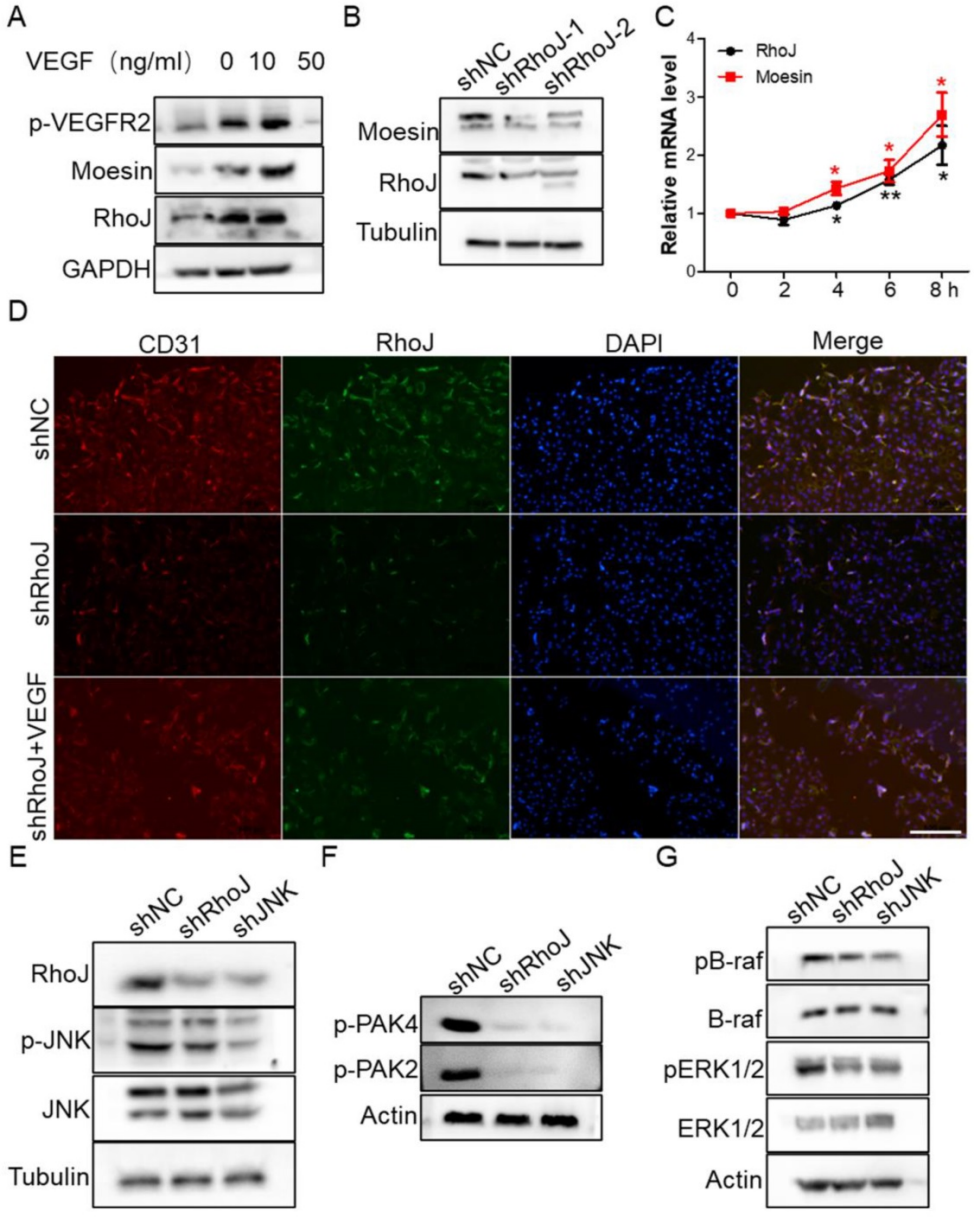
VEGF restores RhoJ silencing-induced CD31 reduction. (A) The expression of RhoJ, moesin and p-VEGFR2 in HUVEC cells stimulated by VEGF at different concentrations were detected by Western blotting. (B) The expression of moesin in HUVEC cells with RhoJ knockdown was detected by Western blotting assay. (C) The mRNA expression levels of RhoJ and moesin in HUVEC cells stimulated by VEGF (50 ng/mL) were detected by qPCR. (D) Representative images for IF analysis of EpCAM and CD31. Scale bar, 100 μm.

To determine the molecular mechanism of VEGF-induced HUVECs migration and sprouting inhibition by SP600125 treatment, we stimulated HUVECs with VEGF to detect whether RhoJ was altered. We found that VEGFR2 was activated expectedly after VEGF treatment, and RhoJ expression level was also upregulated, and the protein level of RhoJ was increased in a VEGF concentration-dependent manner (**Fig. 5A**). Next, we detected the expression of moesin in HUVECs after RhoJ silencing and found that the expression of moesin was also down-regulated when RhoJ was knocked down (**Fig. 5B**). VEGF stimulation increased the mRNA levels of RhoJ and moesin in HUVECs, and their expression levels increased in a time-dependent manner (**Fig. 5C**). Silencing of RhoJ reduced the fluorescence intensity of CD31. Besides, VEGF robustly rescued the expression levels of CD31 and RhoJ in RhoJ stable knockdown HUVECs (**Fig. 5D**).

Previous studies have shown that RhoJ expression is directly regulated by the transcription factor c-Jun, and PAK protein is a key downstream effector for regulating cytoskeletal function among numerous Rho GTPase targets 27. We found that two members of the PAK family are crucial for the angiogenesis of HUVECs. JNK-RhoJ dependent endothelial cell tube formation involves BRAF-ERK signaling. JNK-RhoJ first activates PAK2 and PAK4 and then activates RAF kinase BRAF, leading to the activation of ERK1/2. We found that silencing of RhoJ and JNK significantly down-regulated the activities of PAK2 and PAK4. Similarly, the phosphorylation level of BRAF and ERK1/2 were also significantly reduced after the knockdown of RhoJ and JNK (**Fig. 5E-G**).

**Figure 6:**
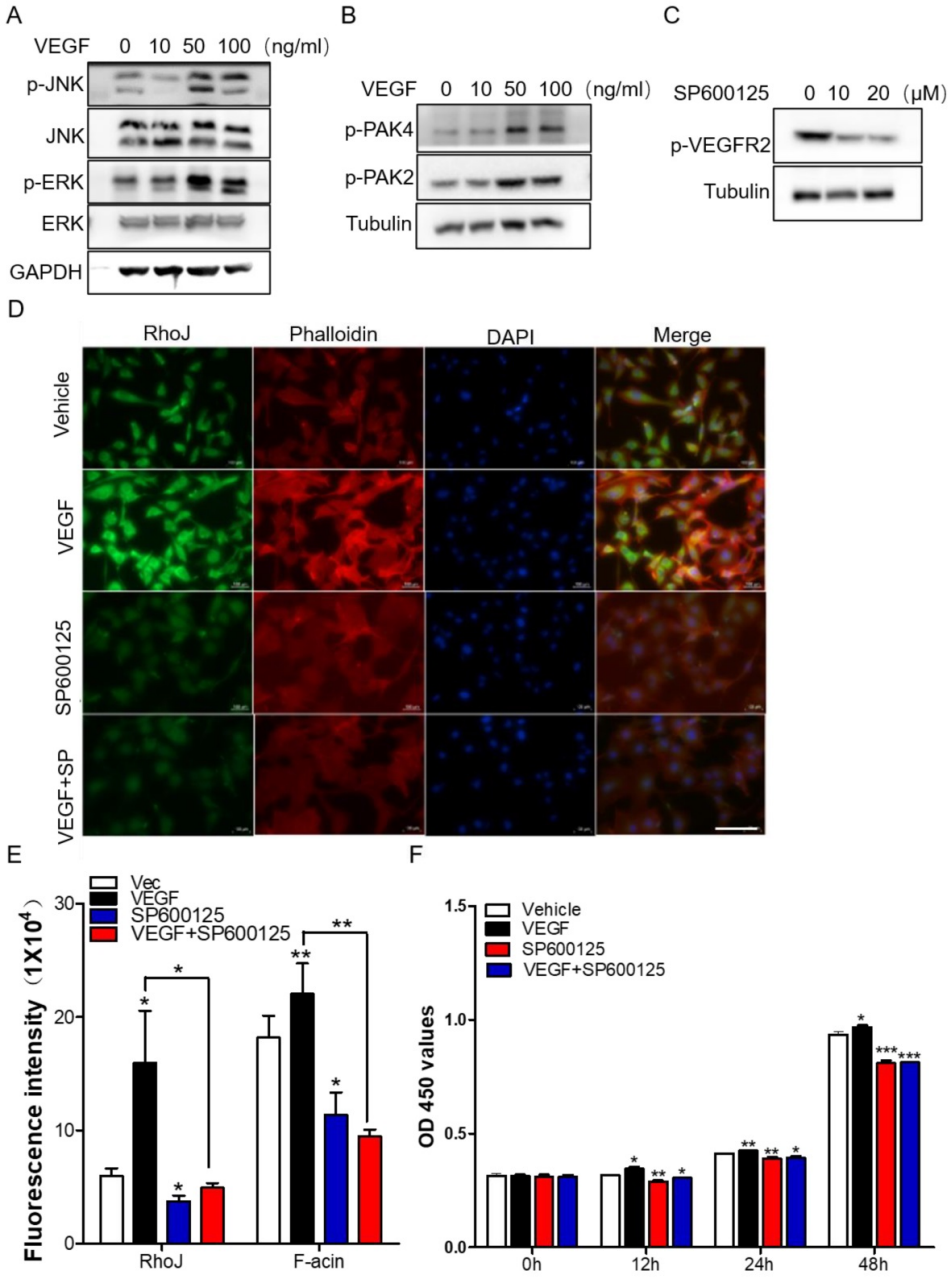
JNK/VEGFR2/RhoJ mediates activation of PAK and ERK pathways. (A) After HUVECs were treated with VEGF at different concentrations, cell lysates were subjected to immunoblotting to detect the activity of JNK and ERK, and (B) PAK2, PAK4. (C) The effect of SP600125 stimulation on VEGFR2 activation in HUVEC cells was detected by Western blotting. (D) Immunofluorescence staining for RhoJ, Pholloidin, and DAPI in HUVECs with or without treatment of VEGF or JNK inhibitor. Scale Bar 100 μm. (E) Quantification of fluorescence intensity of RhoJ and F-actin in (D). Scale bar, 100 μm. (F) the Cell Counting Kit-8 (CCK-8) assays of HUVECs after applying VEGF, SP600125, or vehicle. In all images, data are shown as the mean ± SEM. Every experiment was repeated for three times. *p<0.05; **p<0.01; ***p<0.001.

Subsequently, we stimulated HUVECs with different concentrations of VEGF and collected total proteins for Western blot to detect the activation of JNK/PAK2/PAK4/ERK. Data showed that the level of phosphorylated JNK/PAK2/PAK4/ERK was enhanced in a VEGF concentration-dependent manner (**Fig. 6A, B**). Meanwhile, treatment of HUVECs with JNK inhibitor SP600125 significantly inhibited VEGFR2 activation (**Fig. 6C**). These data demonstrated that there might exist a VEGF-JNK/ERK-VEGF circuitry. Next, we detected the expression of RhoJ and phalloidin-conjugated F-actin by immunofluorescence. We found that the fluorescence intensity of RhoJ was increased after VEGF stimulation but decreased after SP600125 treatment, suggesting that VEGF could not override the down-regulating effect resulting from SP600125 in HUVECs (**Fig. 6D, E**). Then cell growth and viability were measured by CCK8 assay. Consistent with previous findings, VEGF promoted the growth of HUVECs, and SP600125 significantly attenuated the growth, while VEGF failed to resist the growth inhibition caused by SP600125 (**Fig. 6F**). These results showed that RhoJ promoted HUVECs angiogenesis by JNK/VEGFR2 mediated the activation of PAK and ERK pathways.

**Figure 7:**
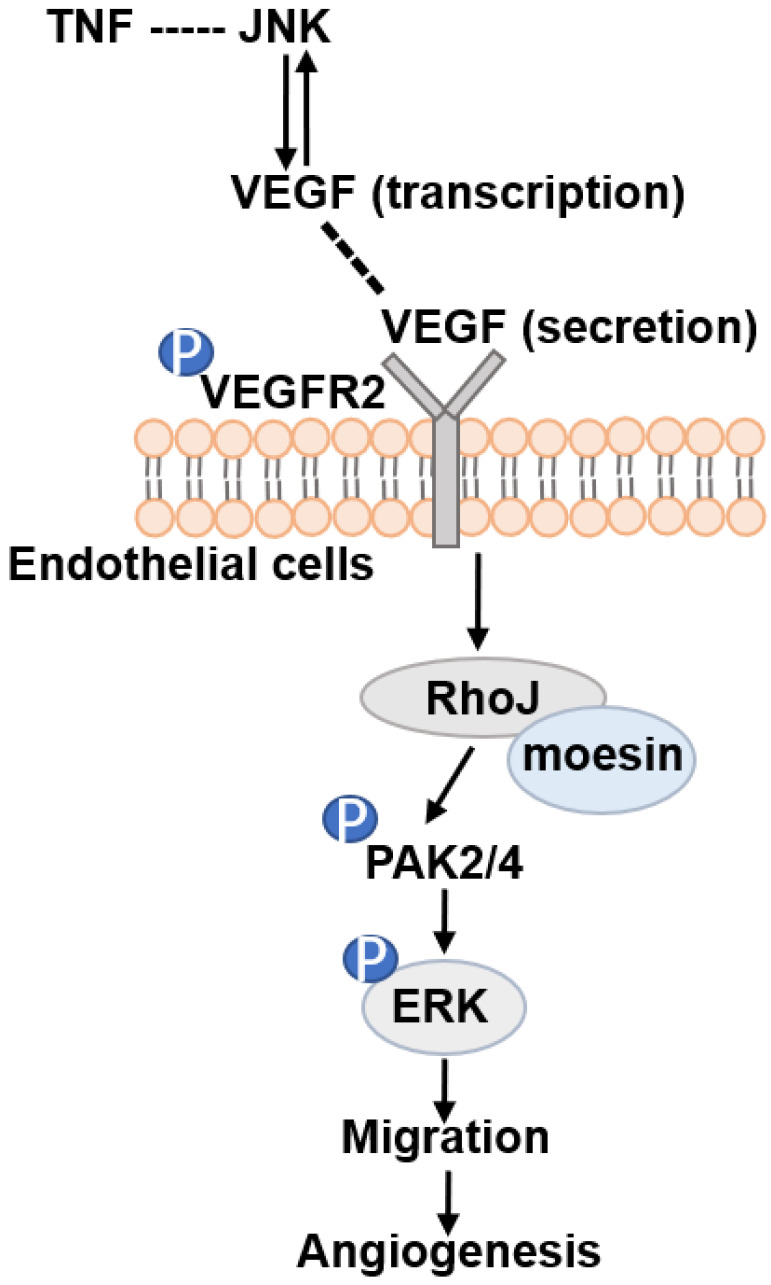
Schematic illustration of JNK-VEGFR2-RhoJ-PAK2/PAK4-BRAF-ERK pathway regulating GBM angiogenesis. JNK inhibitor SP600125 inhibits RhoJ expression level, the phosphorylation level of VEGFR2, and its downstream PAK and ERK1/2 signaling pathways, which regulate endothelial cell function and further impact GBM angiogenesis and progression.

Altogether, our data demonstrate that RhoJ is regulated by JNK/VEGFR2 mediated PAK and ERK pathways in GBM angiogenesis. Activated ERK1/2 and PAK2/PAK4 pathways by RhoJ play an important role in the regulation of HUVECs migration, sprouting and proliferation in angiogenesis of GBM (**Fig. 7**).

## Discussion

GBM is one of the malignant tumors with the worst curative effect. The blood-brain barrier (BBB) in GBM limits cytocidal effect due to the restriction of cytotoxic drug concentration that reaches the tumor sites 28, 29. The BBB prevents the overwhelming majority of molecule-targeted drugs from entering the central nervous system (CNS) 30. At present, the comprehensive treatment method of GBM is still based on surgery, supplemented by radiotherapy and chemotherapy. The chemotherapeutic drug available for GBM is mainly temozolomide (TMZ), which has a poor therapeutic effect. Finding and developing new therapeutic methods for GBM is one of the significant difficulties facing us 31-33.

As early as the 1970s, Folkman et al. found that tumor growth depends on new blood vessel formation providing nutrients and oxygen, and inhibition of angiogenesis may become an important direction of tumor treatment 34, 35. GBM is highly vascularized, and its malignant progression depends on the formation of new blood vessels, and the more severe the vascularization, the higher the malignant degree of GBM and the worse the prognosis 36. Therefore, anti-angiogenic therapy for GBM is a promising therapeutic strategy.

Rho GTPase signaling networks are critical to the vascular structure and tumor angiogenesis 37. The small Rho GTPase RhoJ is abundantly expressed in endothelial cells during tumor progression, and its role in angiogenesis has been extensively reported 38-40. Recent studies have revealed that RhoJ blockade destroys tumor blood vessels by activating the RhoA- ROCK signaling pathway in various tumor models. Xenotransplantation of lung carcinoma and melanoma cells in RhoJ knockout mice and a spontaneous breast tumor mouse model displayed that RhoJ deficiency causes a double attack on tumor blood vessels. On the one hand, it leads to tumor vascular failure by inhibiting tumor angiogenesis and destroying the existing tumor blood vessels. On the other hand, blockade of RhoJ combined with cytotoxic chemotherapeutic drugs, angiogenesis inhibitors, or vascular blockers enhanced their anticancer effects 19. Given the highly vascularized GBM and the key function of RhoJ in angiogenesis, we carried out our present research by inquiring about the role of RhoJ in angiogenesis and HUVECs behavior during GBM progression *in vivo* and *in vitro*.

In our studies, we found that RhoJ overexpression promoted the malignant progression of GBM and angiogenesis. Overexpressed RhoJ significantly facilitated the expression of CD31 in blood vessels. These findings were in line with previous studies. In a study about human vitiligo, Goldstein et al. demonstrated that knockdown of RhoJ impacted epidermal melanocyte migration, differentiation and cell cycle 41. Here, we found that RhoJ deficit in HUVEC cells suppressed the migration of GBM cells by transwell assay with the conditioned medium treatment. An aortic ring angiogenesis model showed that RhoJ promotes directional ECs migration in sprouting vessels, and RhoJ knockout suppresses angiogenesis in retinas 39. These findings, together with our results, reveal that RhoJ is an important molecular target in the pathological and physiological angiogenesis process. Blockade of RhoJ may be a therapeutic direction for cancer and cardiovascular disease treatment.

A recent study by Fukushima et al. focusing on angiogenesis in mice ischemic retinopathy, showed that RhoJ activation could be blocked by VEGF and its activation was restored upon the exposure to Sema3 42. They discovered that VEGF and Sema3E needed RhoJ for promoting ECs migration 39. In contrast to our present study, we found that VEGF restored the inhibition of HUVECs migration, tube formation and sprouting by RhoJ knockdown. The reason for this discrepancy is unknown and may be due to the specificity of the tissue or cell type used.

Our RNA-seq data showed that TNF signaling pathway is positively associated with RhoJ. Interestingly, in our previous work, through database analysis and dual-luciferase reporter gene detection, we concluded that c-Jun is also an important upstream regulator of RhoJ 20. Here, we focused our work on the role of JNK, the downstream effector of the TNF signaling pathway, in the regulation of RhoJ in GBM angiogenesis. We found that RhoJ is a downstream molecule of TNF-JNK, indicating that there exist positive and negative feedback regulations between RhoJ and JNK. We also found that inflammation and immunity pathways were changed due to RhoJ knockdown. At present, there is no relevant report on RhoJ in tumor immunity. The role of RhoJ in GBM immunology may be our subsequent work to do.

In view of the role of VEGF in angiogenesis, we checked the differential expression of VEGF-related genes and found that VEGFA was downregulated in RhoJ knockdown cells. Previous studies have proved that ERK inhibitor PD98059 and JNK inhibitor SP600125 can significantly inhibit VEGF expression in HUVEC cells. Activation of JNK drives the phosphorylation of transcription factor c-Jun/AP-1, thereby inducing VEGF expression 43. Here, we found that VEGF-induced HUVECs migration and sprouting can be inhibited by SP600125 treatment. PAK protein is a key downstream effector of Rho GTPase regulating cytoskeleton function. Two members of the PAK family, PAK2/PAK4, play an irreplaceable role in EC lumen formation in the 3D collagen matrix 44. Moreover, recent studies have shown that after PAK2 and PAK4 were phosphorylated by PKCϵ, or SRC family kinases, SRC and YES, then they induced BRAF/CRAF-ERK1/2 activation in Cdc42-dependent EC lumen formation, and eliminations of RhoJ and ERG significantly reduced the activation of PAK2/PAK4 and BRAF 18. In our study, we indeed discovered that PAK2/PAK4 and ERK were downstream effectors of RhoJ in HUVECs. Silencing of JNK and RhoJ significantly reduced the activation of PAK2/PAK4 and ERK1/2. Recently, the antagonism function of RhoJ in opposition to CDC42 in atherosclerosis in resting vessels was reported 45. However, the relationship between RhoJ and Cdc42 in GBM has not been reported, which is worth studying.

In conclusion, we found that silencing RhoJ could inhibit HUVECs migration ability and HUVEC tube formation ability. Overexpression of RhoJ promoted the expression of vascular endothelial cell marker CD31, epithelial cell adhesion molecule EpCAM, and moesin, suggesting that overexpression of RhoJ promotes angiogenesis and malignant progression of GBM. HUVEC cells treated with VEGF upregulated the expression of RhoJ in a VEGF concentration-dependent manner, indicating that RhoJ is an important downstream signaling molecule of VEGF in GBM angiogenesis. JNK-VEGFR2-RhoJ-PAK-BRAF-ERK pathway plays an important role in the regulation of RhoJ in GBM angiogenesis.

## Supplementary Material

Supplementary methods, figures and table.

## Abbreviations

GBM: Glioblastoma
VEGF: Vascular endothelial growth factor
VEGFR2: Vascular endothelial growth factor receptor 2
VECs: vascular endothelial cells
PAKs: p21-activated kinases
TCL: TC10-Like
HUVECs: Human umbilical vein endothelial cells
LVES: large vessel endothelial supplement
IHC: Immunohistochemistry
OCT: optimal cutting temperature
FBS: fetal bovine serum
DAPI: 4′,6-diamidino-2-phenylindole
IF: Immunofluorescent staining
BEV: bevacizumab/avastin
