# Supplementary material for: RhoJ facilitates angiogenesis in glioblastoma via JNK/VEGFR2 mediated activation of PAK and ERK signaling pathways
Source: Int J Biol Sci. 2022 Jan 1;18(3):942–55. doi: 10.7150/ijbs.65653 (PMC8771846; doi:10.7150/ijbs.65653)
Supplement: Supplementary file 1 — Supplementary methods, figures and table. [file ijbsv18p0942s1.pdf]

RhoJ facilitates angiogenesis in glioblastoma via JNK/VEGFR2 mediated activation of PAK and ERK signaling pathways

Supplementary Materials and methods

Enzyme-Linked ImmunoSorbent Assay (ELISA)

ELISA MAX™ Deluxe Set Human VEGF kit (Biolegend，446504) was used. We collected the supernatant culture medium from U87-shNC and U87-shRhoJ or U87-con and U87-RhoJ-oe cells 24h after seeding. One day prior to the experiment, coated 96-well plate with 100 μL diluted capture Antibody, then the plate was incubated overnight at 4 ℃. On the second day, we brought all the reagents to room temperature (RT), washed the plate 4 times, added 200 μL 1× Blocking Buffer A, incubated 1 h with shaking at RT. Washed 4 times, added 50 μL Assay Diluent D to the standard and sample wells. We added 50 μL diluted standards to the standard wells, added 50 μL samples to the sample wells, incubated 2 h at RT with shaking. We washed 4 times. Added 100 μL diluted Detection Antibody, then incubated 1 h at RT with shaking. Washed and added 100 μL/well of diluted Avidin-HRP solution, sealed plate and incubated at RT for 30 minutes with shaking. Washed and added 100 μL/well Substrate Solution D and incubated in the dark for 10 minutes. Positive wells should turn blue in color. We stopped the reaction by adding 100 μL/well of Stop Solution. Positive wells should change from blue to yellow in color. Read absorbance at 570 nm within 15 minutes.

Supplementary figure legends

Fig. S1 Knockdown of RhoJ inhibits the secretion of VEGF in GBM cells. (A) Transwell assay in HUVECs treated by the conditioned medium from U87 cells with shNC or shRhoJ. The results showed the medium from U87-shRhoJ cells significantly inhibited the migration of HUVECs compared with shNC group. All scale bars, 100 μm. (B) Quantitative analysis of migrated cells in (A). (C) Tube formation assay detected the formed tubes of HUVECs treated with conditioned medium from U87-shNC or U87-shRhoJ. Scale bars, 100 μm. (D) The number of tubes formed was calculated to quantify the ability of tube formation. (E) knockdown of RhoJ significantly decreased the VEGF secretion in U87 cells. (F) knockdown of RhoJ also significantly decreased the VEGF secretion in HUVEC cells. Data are shown as the mean ± SEM. All experiments were performed independently three times. *p<0.05; **p<0.01; ***p<0.001.

Fig. S2 BEV affects RhoJ function on HUVEC migration and tube formation. (A) Transwell assay in HUVECs-con or HUVECs-RhoJ-oe treated by vehicle or Bevacizumab (BEV, 5μg/ml). All scale bars, 100 μm. (B) Quantitative analysis of migrated cells in (A). (C) Tube formation assay detected the formed tubes of HUVECs-con or RhoJ-oe treated with vehicle or BEV (5μg/ml). Scale bars, 100 μm. (D) The number of tubes formed was calculated to quantify the ability of tube formation (con- vehicle group vs RhoJ-oe- vehicle group; RhoJ-oe- vehicle group vs RhoJ-oe+BEV group). Data are shown as the mean ± SEM. All experiments were independently performed three times. *p<0.05; **p<0.01; ***p<0.001.

Supplementary figures

FigS1

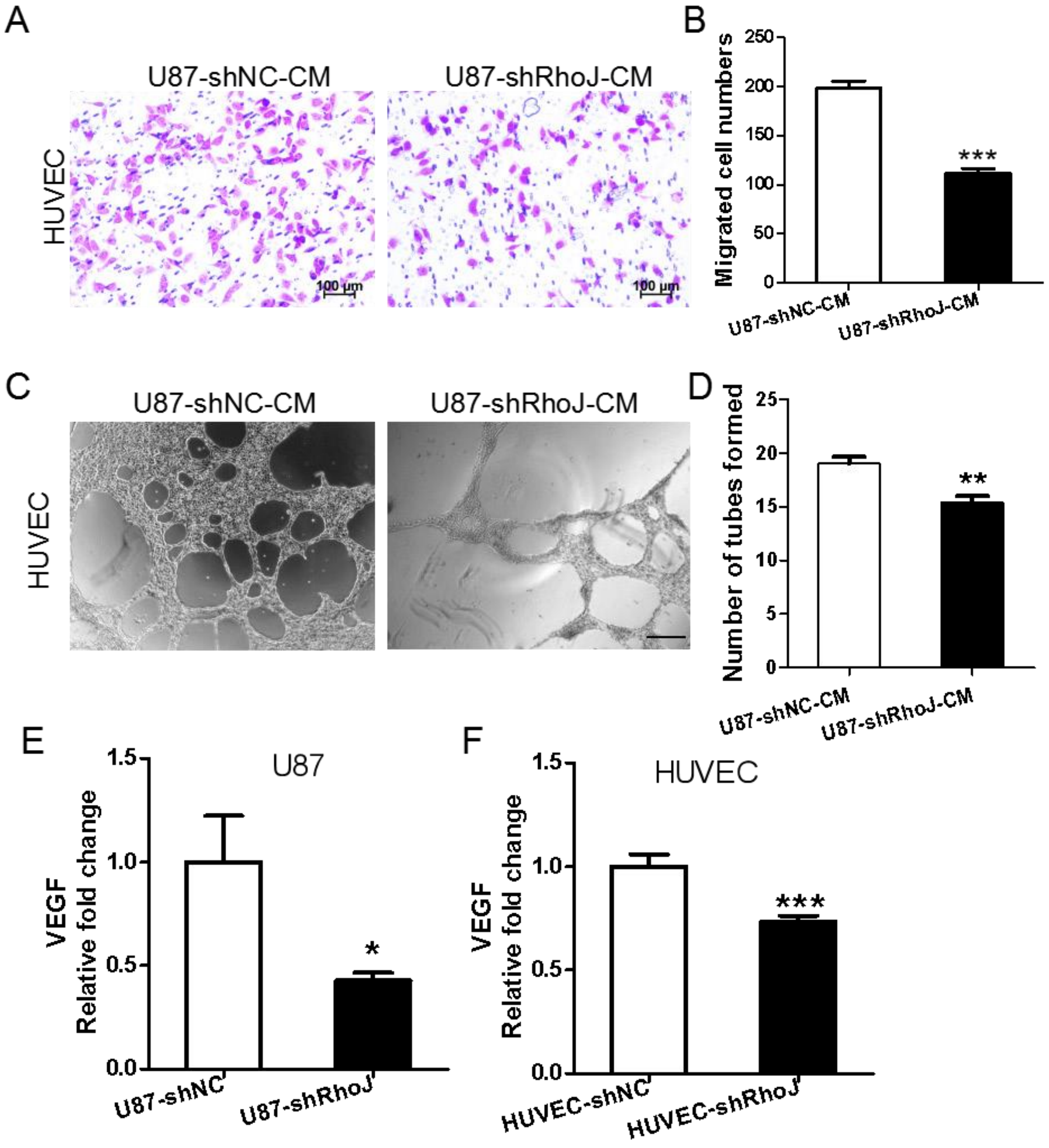

FigS2

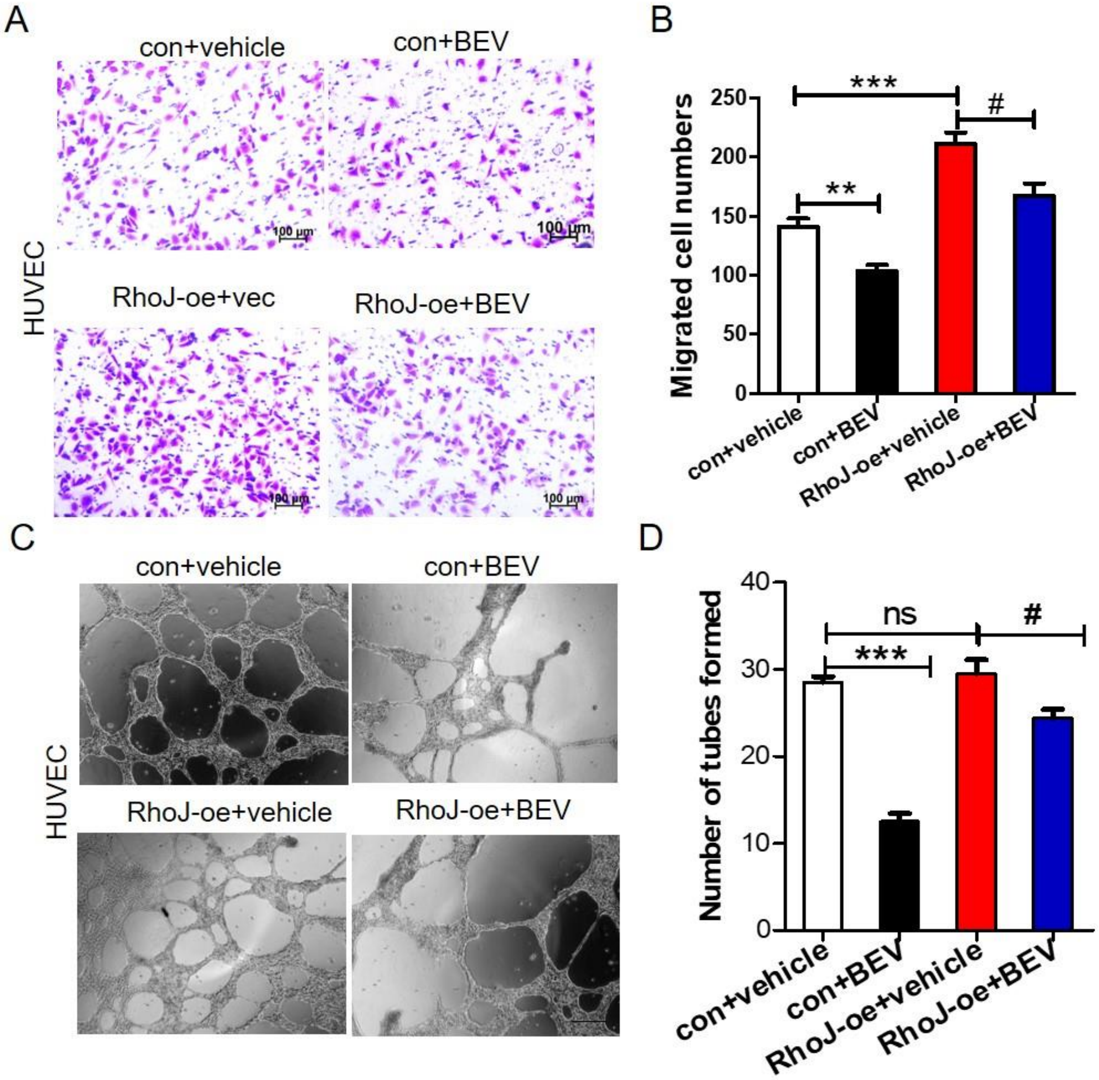
A
con+vehicle
con+BEV
HUVEC
RhoJ-oe+vec
RhoJ-oe+BEV
100 μm
B
Migrated cell numbers
250
200
150
100
50
0
***
#
**
con+vehicle
con+BEV
RhoJ-oe+vehicle
RhoJ-oe+BEV
C
con+vehicle
con+BEV
HUVEC
RhoJ-oe+vehicle
RhoJ-oe+BEV
D
Number of tubes formed
40
30
20
10
0
ns
#
***
con+vehicle
con+BEV
RhoJ-oe+vehicle
RhoJ-oe+BEV

Supplementary tables

Table S1. Primer sequences used in RT-qPCR analysis.

Target	Forward primer (5’-3’)	Reverse primer (5’-3’)
RhoJ	CCTGAGTGACAGAGAAAGAACC	GGAGTGTGTGCGTATGAAAGA
NOD2	CCCTGCAGCTGGACTACAACT	AGATGCCTCGGTCTGAGATATTG
TRAF1	GGAGGCCCAACTGCAATAA	GTCAGCCGTGGGAACAATAA
TNF	GATCCCTGACATCTGGAATCTG	GAAACATCTGGAGAGAGGAAGG
VEGFA	CAGGACATTGCTGTGCTTTG	CTCAGAAGCAGGTGAGAGTAAG
NFkBIA	CATCCTGAAGGCTACCAACTAC	GGCTCCTGAGCATTGACAT
Moesin	GTCAAGTGTGGAGTAGGTTG	CATTCCCTAGACCGCATAAC
GAPDH	CTCCACTCACGGCAAATTCA	GCCTCACCCCATTTGATGTT
